# Nonlinear Propagation and Filamentation on 100 Meter Air Path of Femtosecond Beam Partitioned by Wire Mesh

**DOI:** 10.3390/s22176322

**Published:** 2022-08-23

**Authors:** Yuri E. Geints, Olga V. Minina, Ilia Yu. Geints, Leonid V. Seleznev, Dmitrii V. Pushkarev, Daria V. Mokrousova, Georgy E. Rizaev, Daniil E. Shipilo, Irina A. Nikolaeva, Maria V. Kurilova, Nikolay A. Panov, Olga G. Kosareva, Aurélien Houard, Arnaud Couairon, Andrey A. Ionin, Weiwei Liu

**Affiliations:** 1 V.E. Zuev Institute of Atmospheric Optics, 1 Acad. Zuev Square, Tomsk 634021, Russia; 2 Faculty of Physics, Lomonosov Moscow State University, Leninskie Gory, Moscow 119991, Russia; 3 P.N. Lebedev Physical Institute of the Russian Academy of Sciences, 53 Leninskiy Prospect, Moscow 119991, Russia; 4 LOA, ENSTA Paris, CNRS, Ecole Polytechnique, Institut Polytechnique de Paris, 828 Bd des Maréchaux, 91762 Palaiseau, France; 5 CPHT, CNRS, Ecole Polytechnique, Institut Polytechnique de Paris, Route de Saclay, 91128 Palaiseau, France; 6 Institute of Modern Optics, Tianjin Key Laboratory of Micro-Scale Optical Information Science and Technology, Nankai University, Tianjin 300350, China

**Keywords:** femtosecond filamentation, beam regularization, remote discharge triggering, infrared supercontinuum

## Abstract

High-intensity (∼1 TW/cm${}^{2}$ and higher) region formed in the propagation of ∼60 GW, 90 fs Ti:Sapphire laser pulse on a ∼100 m path in air spans for several tens of meters and includes a plasma filament and a postfilament light channel. The intensity in this extended region is high enough to generate an infrared supercontinuum wing and to initiate laser-induced discharge in the gap between the electrodes. In the experiment and simulations, we delay the high-intensity region along the propagation direction by inserting metal-wire meshes with square cells at the laser system output. We identify the presence of a high-intensity region from the clean-spatial-mode distributions, appearance of the infrared supercontinuum wing, and occurrence of the laser-induced discharge. In the case of free propagation (without any meshes), the onset of the high-intensity zone is at 40–52 m from the laser system output with ∼30 m extension. Insertion of the mesh with 3 mm cells delays the beginning of the high-intensity region to 49–68 m with the same ∼30 m extension. A decrease in the cell size to 1 mm leads to both delay and shrinking of the high-intensity zone to 71–73 m and 6 m, respectively. Three-dimensional simulations in space confirm the mesh-induced delay of the high-intensity zone as the cell size decreases.

## 1. Introduction

Femtosecond filamentation on the atmospheric path [1,2,3] is usually associated with terawatt [4] or even multi-terawatt [5] pulses, whereas the critical power for self-focusing $P_{cr}$ in air is 5–10 GW at the central wavelength of 800 nm [6]. Multiple filaments formed in a TW-peak-power beam emit the supercontinuum or white light [7,8], generate the nitrogen fluorescence [9] from extended plasma channels [10], and can be applied to transport the high-intensity radiation towards a solid [11] or liquid [12,13] target. All these effects provide an efficient tool for the remote sensing of the atmosphere [14,15]. So, the lidar measurements [4] allowed Kasparian et al. to observe the replicas of water absorption lines in the spectrum of the supercontinuum reflected from the clouds at the altitude of 10 km. The fluorescence of the plasma channels in air with impurities was recorded in the spectroscopic studies of the smoke cloud [16] and the alkanole flame [17,18]. This opens up a way for remote combustion diagnostic in engines by a femtosecond filament [19]. The high-intensity filament can excite the fluorescence of atmospheric aerosol. A clean spectrum of water microparticles containing riboflavin was recorded using filament-induced breakdown spectroscopy with the target aerosol cloud located 45 m away [12] from the *Teramobile* laser system [20].

A natural way to control the plasma and white light spatial position in the course of multiple filamentation is the pulse chirping [21,22]. Pulse elongation due to chirping results in the peak power decrease and delay in the filament formation distance. Negative chirp leads to the pulse compression and increase in the overall plasma string extension [21,22]. The transverse spatial modulation of the beam with the power $P\gg P_{cr}$ enables pulse energy segmentation into several sub-apertures, thus providing the transverse regularization of the inherently stochastic multiple filamentation [22,23,24,25,26,27,28,29,30,31,32,33,34,35,36,37]. For example, a “coronary” beam [36,38,39] composed of several coherent sub-beams with $P>P_{cr}$ arranged in a ring moves the filament onset away from the laser system output.

The absorbing periodic meshes were applied to create the two-dimensional periodic structure of the filaments in the beam with the power $P\gg P_{cr}$. The first experiments on multiple filament regularization by means of a mesh were performed in liquids [23,24,40,41]. The square matrix of ∼100 permanent filament-induced refractive index modifications was recorded in the course of geometrical focusing of femtosecond radiation regularized by the mesh into the bulk of the fused silica sample [25]. The mesh was used to regularize stochastic filamentation on the 100 m path in air [30]. In this experiment, multiple filamentation was produced at the output of the hybrid Ti:Sa/KrF sub-picosecond 248 nm laser facility with the pulse peak power of ∼3000 $P_{cr}$. The stable from one laser shot to another formation of a single filament in the center of each mesh cell allowed Shipilo et al. [30] to trace hot spot evolution with the propagation distance and estimate the length of an uninterrupted light filament as more than 15 m.

The pulse chirping or division into sub-beams cannot be applied to filamentation control if the pulse peak power is rather low, e.g., 5–10 times the critical power for self-focusing. Such a peak power range is still of interest for atmospheric applications of filamentation. Indeed, the pioneering experiments on femtosecond laser filamentation in air [1,2,3] were carried out using ∼800 nm pulses with the peak power *P* less than ∼200 GW and provided researchers with the detailed information on the beam distribution [1,3] and conical emission in the visible part of the spectrum [2,42]. In our recent experiments on the 100 m path [43,44,45], the ∼60 GW pulse formed ∼30 cm plasma filament followed by ∼40 m postfilament [22,46,47] with the intensity higher than ∼1 TW/cm${}^{2}$. Such high intensity is sufficient to enhance the infrared spectral wing [43] and to trigger high-voltage discharge [44]. The great advantage of the postfilament channels formed in air under the propagation of a moderate-power beam as compared to a ∼100 TW/cm${}^{2}$ filament is the predictable location of the hot zone along the path. Indeed, in our experiments, the fluctuations of the filament onset estimated in [44] as 3 m are an order of magnitude shorter than the overall postfilament length of about 40 m.

The elaborated approaches of filament intensity measurement [48,49] cannot be trivially transferred to 1–10 TW/cm${}^{2}$ intensity measurement on the extended path. Usually, the evaluation of the postfilament intensity comes from the simulations [43,50] via nonlinear Schrodinger equation (NLSE) [51] or Forward Maxwell equation (FME) [52]. A femtosecond filament evolves in both temporal *t* and spatial (x,y,z) domains [3]. So, a theoretical description of the effect requires, in general, the solution of the four-dimensional t+(x,y,z) mathematical problem [53,54,55]. In the case of the moderate pulse power and the absence of the strong perturbations in the beam transverse distribution, a three-dimensional $t+(r=\sqrt{x^{2}+y^{2}},z)$ problem can be simulated instead. Whereas the beam perturbations are crucial, the transverse (x,y) domain cannot be truncated to one dimension *r*. However, it is possible to average NLSE over the time domain [56] to consider (x,y,z) dynamics only and keep the computational costs affordable. The validity of this (x,y,z) simplified approach is proven for the beam propagation over several Rayleigh lengths [39]. At such distances, one can neglect the effects of the intra-pulse dynamics of the optical intensity (light shock wave, pulse temporal steepening, electron concentration kinetics) and the temporal “memory” of the medium (material dispersion, molecular Raman scattering).

Inhomogeneities on the beam transverse profile with the power lower than $P_{cr}$ can fuse into the lobe with $P>P_{cr}$ during the propagation in the medium with cubic nonlinearity [57]. Such interaction between the sub-beams results in the delay of the filament onset [58]. The filamentation in air of the beam with two perturbations was investigated in the experiments [59,60] on the several-meter path. In [60], two replicas of the beam formed by the beam splitter were sent to two arms of the same length and then combined on another beam splitter with ∼1 mm transverse offset. Beam combining was done simultaneously with fine temporal overlap between the two arms, i.e., the experimental technique was rather complicated and, therefore, cannot be directly transferred onto an atmospheric path.

In general, any amplitude mask or phase plate can divide the beam onto several sub-beams, i.e., organize inhomogeneities on the beam transverse profile. However, the phase plates usually inhibit the interaction between inhomogeneities and their fusion into a single filament due to the π-phase break between the sub-beams [27,35,61]. So, the amplitude masks, e.g., meshes at the laser system output seem to be preferable for the filamentation delay due to the interaction between the sub-beams, each of which has the power below the critical power for self-focusing.

**Figure 1:**
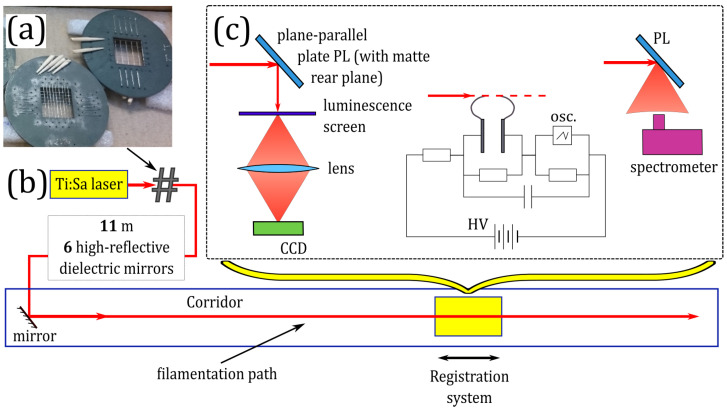
(**a**) Photo of wire meshes used for the beam regularization. Scheme of (**b**) experimental setup and (**c**) portable registration system. The latter allowed us to record the transverse fluence distributions (**left**), check the discharge triggering (**center**), and record the pulse spectrum (**right**). The symbol “#” in (**b**) indicates the mesh inserted into the beam. In (**c**), PL is a plane-parallel plate with a single matted surface, HV is a high-voltage supply, Osc is an oscilloscope.

The purpose of this work is to control the longitudinal location of the high-intensity zone including the plasma filament and light postfilament by inserting the meshes with 1 or 3 mm square cells (see Figure 1a) into the beam at the output of ∼60 GW Ti:Sapphire laser system. We identified the high-intensity (∼1 TW/cm${}^{2}$ and higher) zone in the 100 m corridor from the transverse beam distributions, the spectra of infrared supercontinuum [43], and the possibility of laser-induced discharge [44] at a set of different distances along the propagation path. In the case of free filamentation (without mesh at the laser system output), the high-intensity zone starts at 40 m and spans to 80 m. The insertion of the 1 mm mesh results in the shift of the high-intensity zone to 71–77 m. Since our experimental diagnostics provides only indirect estimations of the pulse intensity, we support the identification of the high-intensity zone with (x,y,z) simulations of nonlinear beam propagation and filamentation.

## 2. Materials and Methods

### 2.1. Experiment

Experimental studies on filamentation of laser pulses modulated in the transverse plane in air were carried out the using commercial Ti:Sa laser system (Avesta ltd.), delivering pulses with the central wavelength $\lambda_{0}=744$ nm, the full width at half-maximum (FWHM) duration of ∼90 fs, and the energy of ∼6.2 mJ at 10 Hz repetition rate. Initially, a slightly elliptical laser beam with the diameter of ∼8 mm was directed to the ∼100 m optical path in air inside the building corridor using seven high-reflective mirrors. The last mirror was located at a distance of about 11 m from the laser system output, see Figure 1b. To avoid self-focusing collapse within the first 11 m of the optical path, the system of mirrors introduced a slight divergence into the laser beam with the equivalent focal distance f≈−50 m, see the details in [43].

In our experiments, we regularized the beam using handmade metal wire meshes with 1 and 3 mm square cells (Figure 1a). To minimize the pulse energy absorption, we made our meshes from nichrome 100 μm wires. The pulse energy dropped down to 6 and 5 mJ after the mesh with 3 mm and 1 mm cells, respectively. The meshes were placed inside the beam path at the distance of ∼1 m from the laser system output.

In all three cases (free propagation, 1 and 3 mm cell meshes), we identified the position and elongation of the high-intensity zone. Along the propagation coordinate *z*, we recorded the transverse fluence distributions by the CCD-camera Ophir Spiricon SP620U, pulse spectra by a spectrometer Avesta ASP-150, and monitored the possibility to trigger a high-voltage discharge (Figure 1c). During the measurements of the beam distribution and pulse spectrum, we reflected the beam from the plane-parallel plate (PL) with one opaque surface to avoid the damage of the CCD camera and the spectrometer. We directed to the spectrometer radiation reflected from the opaque surface of the PL, thus providing registration of the spectrum integrated over the beam. For the beam visualization, the laser radiation was reflected from the smooth surface of the PL onto a luminescent screen while the opaque surface prevented Fresnel reflection from the rear surface of PL. The luminescence was projected to a CCD camera by an objective lens. Technical details on measurement techniques used can be found in [45].

The discharge triggering system was similar to the one in [44] and consisted of two needle steel electrodes supplied with the direct voltage slightly below 5 kV. The laser pulse propagated parallel to the line connecting the tips of the electrodes separated by 4–5 mm and hit the cathode. An oscilloscope detected the current in the discharge circuit and signal from the photodiode, which responded to the light scattered from a paper screen in the beam path behind the electrodes. The electric discharge was considered as the laser-driven one if the discharge current was synchronized with the photodiode signal.

**Figure 2:**
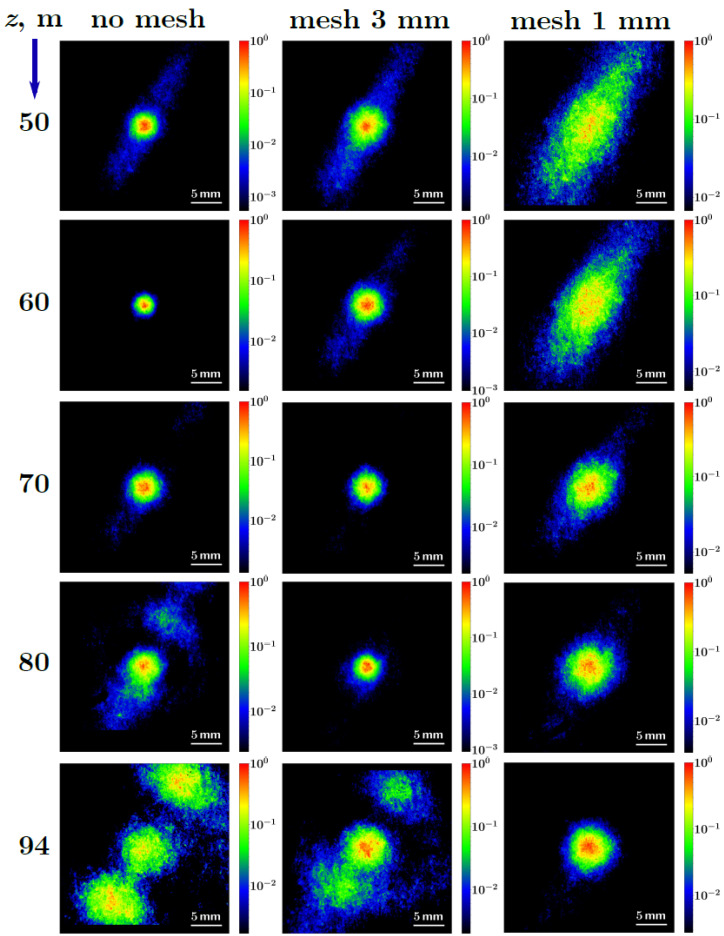
Normalized transverse fluence distributions measured at different distances *z* in the range from 50 m to 94 m for the free propagation without a mesh (left column) as well as in the case of regularization by the mesh with 3 mm cells (middle column) and 1 mm cells (right column).

Figure 2 shows typical transverse fluence distributions of the radiation, each of which is normalized to its maximum, at several distances *z* in the range from 50 m to 94 m in the case of free (left column) and regularized by meshes with 3 mm (middle column) and 1 mm (right column) cells propagation. A relatively clean circular beam mode was observed at the distance z≈60 m for a free beam. When the beam was regularized by mesh with 3 mm cell size, a clean beam mode appeared at the distance of about 70 m. In the case of 1 mm sell size mesh regularization, the beam mode “cleaning” started at the end of the propagation path ∼94 m. So, the beam regularization by the mesh leads to the shift of the distance of circular beam pattern formation. The smaller the mesh cell size, the further in the propagation path the circular beam pattern is formed.

### 2.2. Simulations

To simulate the propagation and filamentation of the beam after the mesh, we used the reduced (x,y,z) version [56] of t+(x,y,z) nonlinear Schrodinger equation (NLSE) [51] for the envelope *E* of the pulse electric field. The initial beam profile E(x,y,z=0) is assumed to be Gaussian with the convex wavefront in accordance with the weak beam defocusing in the experiments (f=−50 m). The non-ideal beam quality is introduced by superimposing random amplitude noise on the initially smooth beam profile: $E\left(x,y\right)\to E\left(x,y\right)\left[1+0.05\times \xi (x,y)\right]$, where ξ(x,y)∈[−1;1] is the random function with normal distribution. The simulations are carried out for the laser beam with the central wavelength $\lambda_{0}=744$ nm, initial radius (at $e^{-1}$ intensity level) of 4 mm and pulse energy of 6.2 mJ before the mesh. The influence of the mesh is simulated by multiplication of the envelope E(x,y) by a periodic amplitude mask Φ(x,y) with the square transparent cells (with the side length of 3 or 1 mm) separated by the opaque narrow boundaries, which model the nichrome wires with the diameter of 100 μm.

**Figure 3:**
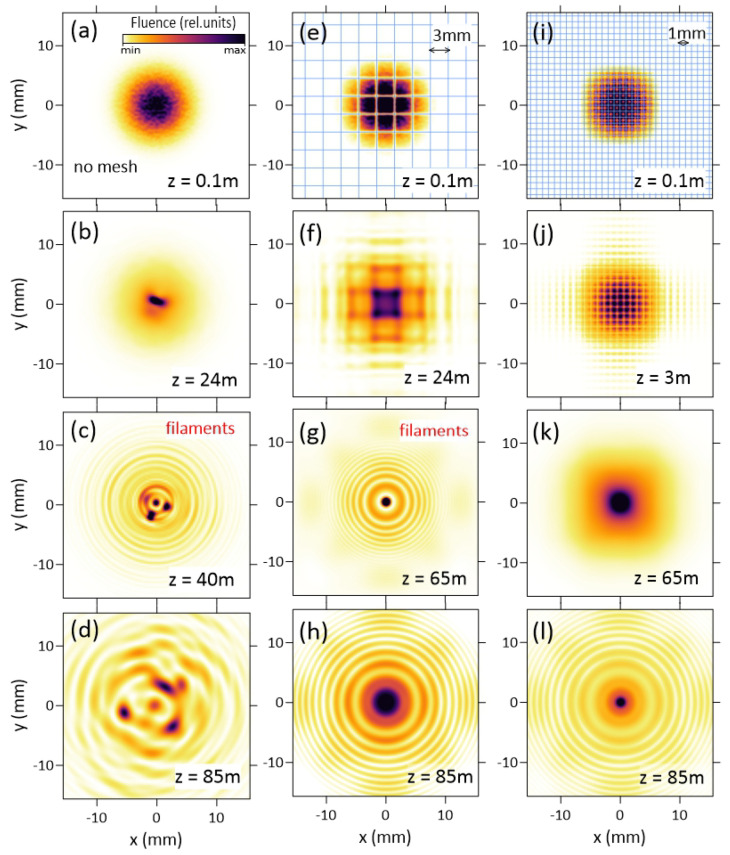
Transverse fluence profiles (normalized to the maximum value) at various propagation distances *z* without modulation (left column, (**a**–**d**)), when modulated by meshes with 3-mm (middle column, (**e**–**h**)) and 1-mm (right column, (**i**–**l**)) cells. Mesh amplitude masks are shown by the blue grids at (**e**,**i**).

The evolution of the transverse profiles of the laser beam fluence with propagation distance *z* is shown in Figure 3, the left, middle and right columns correspond to the free propagation without mesh, 3 mm mesh and 1 mm mesh, respectively. Each fluence distribution shown in Figure 3 is normalized to its maximum. In the case of free propagation, the self-focusing squeezes the beam at the initial stage of propagation z≲24 m. This effect is clearly pronounced despite beam defocusing f=−50 m (cf. Figure 3a,b). In contrast, at the same initial stage, the effect of linear diffraction is significant for the beam after the mesh. First, the change between minima and maxima similar to the Talbot diffraction is clearly seen for the 3 mm cell (cf. Figure 3e,f). Second, the laser beam transverse modulation by meshes causes considerable increase in the global beam divergence as compared to the free propagation due to the strong diffraction on the mesh wires. For example, at z=24 m, the free propagated beam is localized within an area of 14 mm in diameter (Figure 3b), while in the case of the 3 mm mesh, the radiation is distributed within a circle of 24 mm in diameter (Figure 3f).

The Kerr phase shift accumulates with the distance *z*, resulting in the beam mode cleaning (Figure 3k) and the beam collapse at z≈40, 65 and 85 m in the case of free propagation, 3 mm mesh, and 1 mm mesh, respectively, see Figure 3c,g,l. Then, the strong-field ionization stops self-focusing. Diffraction of laser radiation on the self-induced plasma obstacle results in the appearance of multiple divergent rings in the beam profile and in the overall increase of beam divergence as compared to the filamentation region (Figure 3d,h). So, in the case of the mesh with 1 mm cells, at the distance of 85 m, the beam is noticeably smaller than in the case of the mesh with 3 mm cells, cf. Figure 3h with l. Hence, the region of the smallest beam size is shifted along the propagation path further away from the laser system output if the beam is regularized by the mesh with the smaller cell.

## 3. Results and Discussion

In this section, we discuss in details the possibility to control the location of the ∼1 TW/cm${}^{2}$ zone including the filament and the postfilament channel along the propagation path by insertion of the meshes with different cells into the beam at the laser system output. We identify this zone by the minimal beam diameter and the low divergence of laser radiation [43], the high-voltage discharge triggering [44], and the emission of the infrared supercontinuum with the strongly modulated spectrum [43,45]. None of the three diagnostics methods can identify the presence of the laser plasma nor quantify the intensity of the pulse. The discharge triggering seems to occur wherever the pulse intensity exceeds ∼0.5 TW/cm${}^{2}$ yielding only a lower intensity boundary [44]. The spectrum transformation is a “delayed” complex effect which requires propagation with high intensity and cannot indicate the start of the high intensity zone [62]. The cleanest and finest fluence distributions were registered when the spatial confinement of the radiation in the filament took place and the beam cleaning effect had been developed [46,63]. With all this in mind, the three methods combined still lack quantitative characterization, but nevertheless provide a holistic picture of the filamentation scenario.

**Figure 4:**
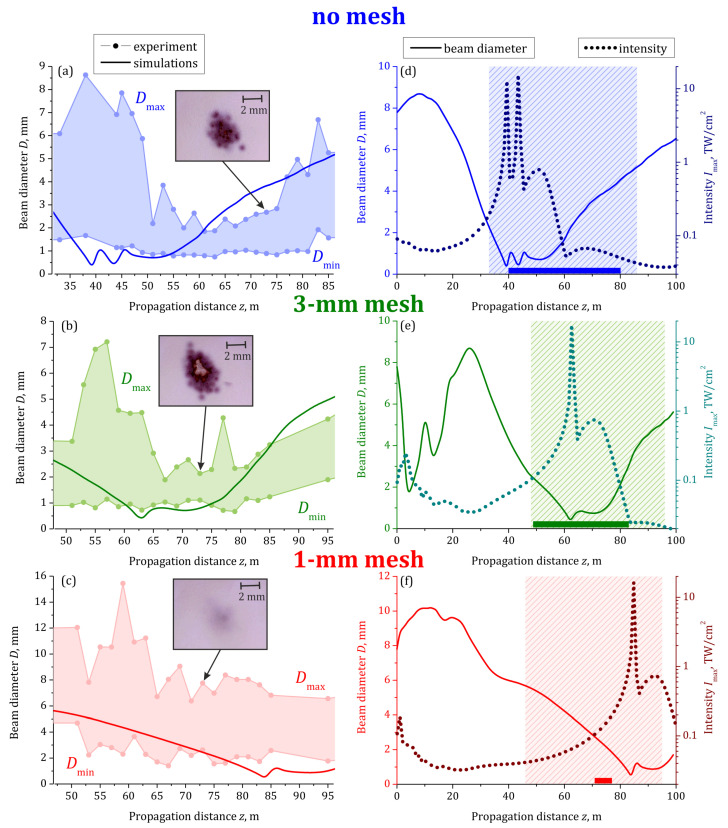
Dependencies of minimal $D_{\min}$ and maximal $D_{\max}$ measured out of 16 laser shots beam diameters (circles) on the propagation distance *z* in comparison with the beam diameter obtained from simulations (solid curves) for different modulation regimes: without modulation ((**a**), blue), using 3 mm ((**b**), green) and 1 mm ((**c**), red) meshes. Sixteen measured at the certain propagation distance *z* diameters lie between $D_{\min}$ and $D_{\max}$ inside the filled area in (**a**–**c**). Insets in the left column show images of burns on the photographic paper at z≈73 m. (**d**–**f**) Dependencies of beam diameter (solid curves) and maximal intensity (dotted curves) on propagation distance *z* obtained in simulations in the range of *z* from 0 to 100 m for all three cases. Colored rectangles highlight the regions shown in left column. Color bands in lower part of (**d**–**f**) demonstrate the range where the laser pulse triggered the electric discharge.

To obtain the dependence of the beam diameter *D* on the propagation distance *z* in the experiment and simulations, we take *x*- and *y*-slices of the fluence distributions, which are crossed in the beam “mass center”, and fit them by the Gaussian functions. From these fits, we extract the two beam widths at $e^{-1}$ and consider the average of them as the diameter *D*. The solid curves in Figure 4 shows the simulated dependencies D(z). The beam diameter in the experiment fluctuates from one laser shot to another. So, the circles in Figure 4a–c represent the minimal $D_{\min}$ and maximal $D_{\max}$ diameters obtained over 16 laser shots at a certain position *z*. The shaded area between the measured dependencies $D_{\min}\left(z\right)$ and $D_{\max}\left(z\right)$ indicates all the observed values of the diameter *D*.

The simulated dependencies of the diameter *D* on the distance *z* are in reasonable agreement with the measured ones. Indeed, the dependencies D(z) obtained from the simulations lie mainly within the shaded area. The length of the postfilament channel with the minimal beam divergence over ∼20 m is clearly seen in the case of the free beam propagation (Figure 4a) and in the case of 3 mm mesh (Figure 4b). Both the experiment and the simulations demonstrate the increase in the *z*-location of the zone with minimal diameter with the decrease in the cell size. In the simulations, this zone starts from 38, 63, and 85 m in the case of the mesh absence, 3 mm mesh and 1 mm mesh, respectively. The experiment reveals a slightly further start of this zone: 52 and 68 m for the mesh absence and 3 mm mesh, respectively. The measured beam diameter for the 1 mm mesh decreases monotonically up to 95 m. So, identification of the minimal-diameter zone for the 1 mm mesh was not possible.

The experiment demonstrates qualitatively that the high-intensity region shift accompanies the shift of the zone with minimal diameter. For this purpose, we inserted the photopaper into the beam for 1 min (600 shots) at the same distance of z≈73 m in all three cases, see insets in Figure 4a–c. The brightest burns on the photopaper appear for the 3 mm mesh for which the mean diameter at the same distance is about 1.5 mm. In the absence of the mesh, the beam exhibits signs of divergence already at 73 m distance; so, the mean diameter is lager than ∼2 mm and the burns are slightly weaker. In the case of the 1 mm mesh, the beam continues to converge exhibiting the mean diameter of ∼5 mm, so the burns are faintly distinguishable.

The simulations confirm that the zone of the minimal diameter is in excellent agreement with the region of the intensity of ∼0.5 TW/cm${}^{2}$ and higher, see Figure 4d–f. Such intensity was estimated in [44] as suitable for high-voltage discharge triggering. To identify the possibility of the pulse to trigger the discharge, we illuminated the cathode of the needle-tip electrodes under the voltage slightly below 5 kV by the laser pulse, similarly to [44]. We checked if the discharge current detected by the oscilloscope was synchronized with the signal from the photodiode. The triggering was observed in all three cases studied. For the reference experiment without a mesh, triggering occurred when the electrodes were placed at any position between 40 and 80 m of propagation. This range is indicated as a horizontal bar in Figure 4d. The insertion of the 3 mm mesh into the beam leads to the displacement of both the start and the end of this zone to 49 and 83 m, respectively, see the horizontal bar in Figure 4e. The discharge triggering probability out of 50 laser shots in these two cases is 60–80%, at any distance inside the triggering region. For the 1 mm mesh, the triggering zone was shortened almost by an order of magnitude and moved away from the laser. The discharge was observed with the probability less than 50% when the electrodes were located at the distance range from 71 to 77 m, see Figure 4f. The observed regions of the laser-induced discharge are satisfactorily represented by the simulated positions of the filament formation: 40, 62, and 85 m in the cases of the mesh absence, 3 mm mesh, and 1 mm mesh, respectively, cf. dotted curves with bars in Figure 4d–f.

**Figure 5:**
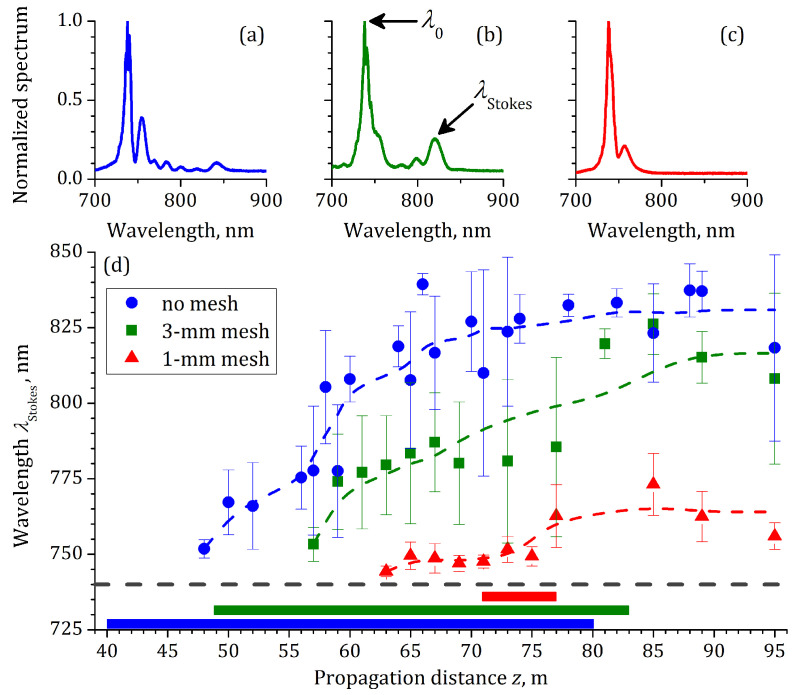
Normalized pulse spectra measured at z=94 m for different modulation regimes: without modulation ((**a**), blue), using 3 mm ((**b**), green) and 1 mm ((**c**), red) meshes. (**d**) Dependencies of the position $\lambda_{\mathrm{Stokes}}$ of the longest-wavelength maximum in the spectrum on the propagation distance *z* for all three cases. Dashed line in (**d**) indicates pulse central wavelength $\lambda_{0}$. Color bands in lower part of (**d**) show the range where the laser pulse triggered the electric discharge.

Another method to identify the high-intensity zone is the measurement of the supercontinuum spectra at the different distances *z* along the propagation path. In our previous work [43], we observed the consistent formation of the infrared humps in the radiation spectra of the postfilament channel. The examples of the multi-hump spectra recorded at z=94 m without a mesh, with 3 mm mesh, and 1 mm mesh are shown in Figure 5a–c, respectively. That the broadband supercontinuum was strongly modulated in the infrared part of spectrum is clearly seen in three cases studied. We measured the spectra at different propagation distances *z* and obtained from each spectrum the wavelength $\lambda_{\mathrm{Stokes}}$ of the most long-wavelength hump, see Figure 5b. The dependencies $\lambda_{\mathrm{Stokes}}\left(z\right)$ for the three cases studied are shown in Figure 5d. The infrared humps start to emerge at z=48, 57 and 73 m in the cases of the mesh absence, 3 mm mesh, and 1 mm mesh, respectively. These *z*-positions are slightly further than the onsets of the discharge triggering zone, cf. markers and bars of the same color in Figure 5d. Nevertheless, they demonstrate the same behavior: the delay of the beginning of the high-intensity zone is associated with the decrease of the cells in the mesh.

**Figure 6:**
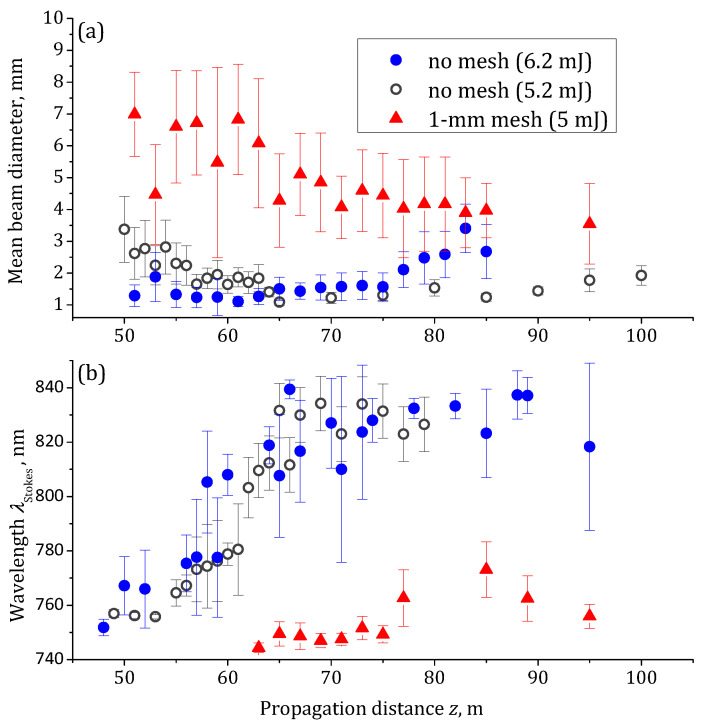
Dependencies of (**a**) the beam diameter and (**b**) the position $\lambda_{\mathrm{Stokes}}$ of the longest-wavelength maximum in the spectrum on the propagation distance *z*. Filled blue circles and open gray circles stand for the free beams with the pulse energies of 6.2 and 5.2 mJ, respectively. Red triangles represent data in the case of beam regularization by the 1 mm mesh.

In principle, the observed shift of the high-intensity zone can be caused by the pulse peak power decrease due to the absorption induced by the mesh. We studied the influence of the pulse energy on the high-intensity zone localization by decreasing the pulse energy down to 5.2 mJ, i.e., approximately the amount remained after 6.2-mJ pulse passes through the 1 mm mesh. We sent the free 5.2 mJ beam into the corridor and measured the dependencies on the distance *z* of the beam diameter *D* and the wavelength $\lambda_{\mathrm{Stokes}}$ of the most long-wavelength hump. The dependencies D(z) and $\lambda_{\mathrm{Stokes}}\left(z\right)$ are almost identical for the free propagation of the 5.2 and 6.2 mJ pulses, cf. open gray and filled blue circles in Figure 6, while the ones for the 1 mm mesh regularization differ from the case of the free beams dramatically, see red triangles in Figure 6. Therefore, the segmentation of the beam plays a dominant role as compared to the energy loss.

## 4. Summary and Conclusions

In conclusion, we have studied experimentally and numerically the nonlinear propagation and filamentation on the ∼100 m path of 744 nm, ∼60 GW, 90 fs, 8 mm in diameter pulse, whose transverse profile is modulated by the mesh with the square cells of 1 or 3 mm side length. In our experiment, we have identified the location of the high-intensity (∼1 TW/cm${}^{2}$ and higher) region, including the filament and the postfilament using three experimental techniques: (i) recording the beam profile on CCD and search for the distance of the minimal beam diameter, (ii) determination of the region of laser-induced discharge, and (iii) measurement of the beam spectra and the estimation of the distance at which the first separated infrared spectral hump emerges. The location of the high-intensity region along the propagation obtained by three experimental methods as well as in numerical simulations is summarized in Table 1. The reasonable agreement between the experimental and simulated distances of the high-intensity region clearly shows the trend of the high-intensity region shifting away from the laser system output with the decrease in the mesh cell size. The possibility to control the start of the high-intensity zone through inserting various meshes while leaving the overall geometry of the experiment and pulse parameters unchanged is important for remote sensing and discharge initiation applications.

## Figures and Tables

**Table 1:** The location of the high-intensity zone along the propagation path obtained by different methods.

Method	Distance (m)		
	No Mesh	3 mm Mesh	1 mm Mesh
(i) Minimal beam diameter	52	68	-
(ii) Region of laser-induced discharge	40–80	49–83	71–77
(iii) Onset of infrared humps emergence	48	57	73
Simulations	40	62	85

## Data Availability

Data underlying the results presented in this paper are not publicly available at this time but may be obtained from the authors upon reasonable request.
